# Prevention of Cardiac Implantable Electronic Device Infections: A Review

**DOI:** 10.31083/j.rcm2406176

**Published:** 2023-06-14

**Authors:** Grzegorz Sławiński, Maciej Kempa, Andrzej Przybylski

**Affiliations:** ^1^Department of Cardiology and Electrotherapy, Medical University of Gdańsk, 80-210 Gdańsk, Poland; ^2^Club 30, Polish Cardiac Society, 00-193 Warsaw, Poland; ^3^Medical College, University of Rzeszow, 35-310 Rzeszów, Poland; ^4^Cardiology Department with the Acute Coronary Syndromes Subdivision, Clinical Provincial Hospital No 2, 35-301 Rzeszów, Poland

**Keywords:** cardioverter-defibrillator, pacemaker, infections, complications

## Abstract

The importance of cardiac implantable electronic devices (CIEDs) in the treatment of cardiac rhythm disturbances, heart failure, and the prevention of sudden cardiac death is indisputable. However, CIED therapy is associated with complications, among which infections are particularly unfavourable in terms of prognosis. The diagnosis and management of CIED infections remain complex, with a significant impact on mortality and healthcare costs. For these reasons, the risk factors for CIED infections and methods of their prevention have been assessed in recent years. This review summarises the current state of knowledge on the subject. We also outlined the role of alternative methods, such as subcutaneous defibrillators, leadless pacemakers, and wearable cardioverter defibrillators.

## 1. Introduction

Indications for the implantation of cardiac implantable electronic devices (CIEDs) are becoming increasingly extensive, which has significantly increased the number of patients with these devices. Apart from classic pacemakers (PM) and implantable cardioverter-defibrillators (ICDs), more complex systems, such as cardiac resynchronisation therapy devices (CRT), are also being implanted more. Over a million CIEDs are implanted each year [1]. The increasing number of CIEDs used and their complexity are unfortunately associated with a growing number of complications, among which CIED infections are particularly unfavourable in terms of prognosis [2]. The incidence thereof is estimated at 0.5%–2.2%, depending on the definitions used, patient populations, and types of implanted devices [3]. CIED infections severely impact both mortality and quality of life [4]. The most serious prognosis concerns patients with a severe CIED infection, for instance, accompanied by septic shock. In these cases, in-hospital mortality is up to 50% [5]. Infective complications, in addition to an unfavourable prognosis, are associated with significant financial burdens for healthcare systems. In their study, Romanek *et al*. [6] evaluated the costs of treatment of patients with CIED infections in Poland, showing that the average cost of therapy for this type of patient is EUR 8010 (1 EUR = 1.07 USD), while for patients with implanted CRT devices, the costs increase to EUR 11,440. For these reasons, the risk factors for CIED infections and the methods of their prevention have been assessed worldwide in recent years. This review summarises the current state of knowledge on the subject.

## 2. Pathophysiology and Etiology

**Fig. 1.**
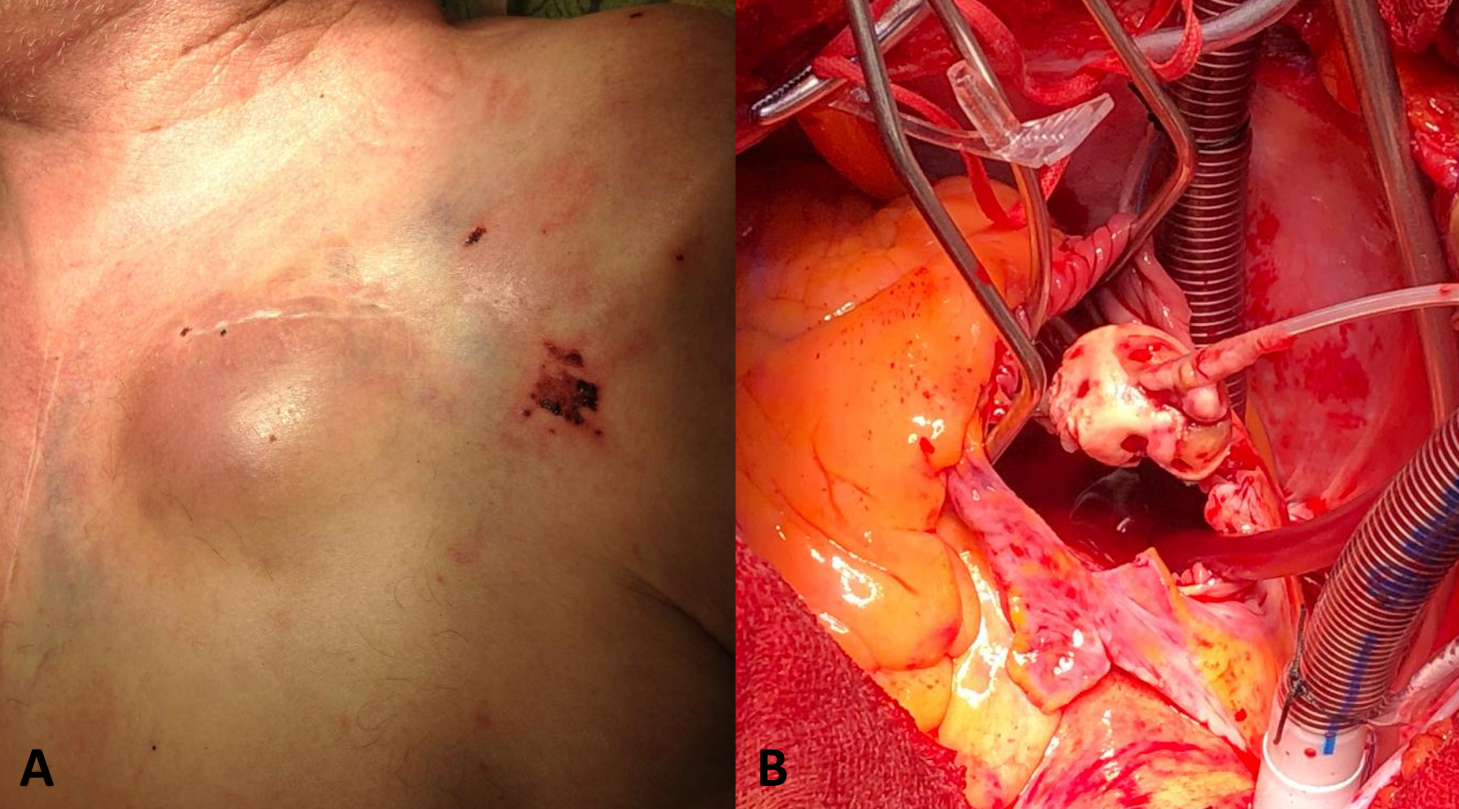
**Clinical manifestations of CIED infections**. (A) Pocket infection. (B) Image from lead extraction showing cardiac device-related infective endocarditis. CIED, cardiac implantable electronic device.

There are two basic ways for CIED colonisation by bacteria. The first one takes place directly during the CIED procedure (implantation *de novo*, replacement, upgrade) and results from direct exposure to microorganisms colonising the patient’s skin. In this situation, the first manifestation of infection is usually device pocket infection, and the involvement of the leads is secondary. The second is the hematogeneous route—colonisation of the intracardiac and intravascular parts of CIED leads at the first stage—which is a complication of infections located in distant places. In this case, the patient presents symptoms of a generalised infection from the very beginning, and the pocket of the device may look completely normal. The dominant manifestation among CIED infections is pocket infection (69% of all infectious complications), and implanted leads are less frequently involved (Fig. 1) [7]. By far, the most common (70%–90%) etiological factors of CIED infections are Gram-positive bacteria—Staphylococcus aureus (30.8%) and coagulase-negative staphylococci (37.6%). Due to the colonisation of the skin by these microorganisms, they are the main cause of early infectious complications in the form of pocket infection. Significantly less frequent are other Gram-positive and -negative bacteria [8]. Methicillin-resistant staphylococci (both coagulase-negative and -positive) account for approximately one-third of all cases [9]. Certain clinical situations are predisposed to bacteremia caused by specific microorganisms. Patients with colon diseases are prone to Gram-negative bacteria infections. In patients with central venous catheters hospitalised in an intensive care unit, coagulase-negative staphylococci, methicillin-resistant Staphylococcus aureus (MRSA), and Gram-negative bacteria P. aeruginosa, K. pneumoniae, E. coli, Enterobacter spp., A. baumannii, and P. mirabilis might be infectious factors. Catheter-associated urinary tract infections, which can result in bacteremia and CIED infections, are commonly caused by E. coli, Enterococci spp., S. aureus, P. aeruginosa, P. mirabilis, and Candida spp. Patients with ventilator-associated pneumoniae should be expected to be cultured positive for P. aeruginosa, members of the family Enterobacteriaceae, A. baumannii, Stenotrophomonas maltophilia, and MRSA [10]. Less common pathogens causing CIED infections are B. melitensis, S. paucimobilis, and K. schroeteri. Brucella is primarily endemic in developing countries, and neurologic and articular symptoms may be present, in addition to generalised infection. S. paucimobilis, a Gram-negative bacillus found in the wood chips of coniferous trees, is a rare cause of opportunistic infection. K. schroeteri is a relatively novel species for which data are limited. However, it has been proven that apart from bacteriemia, it can also cause infections of prosthetic valves [11]. CIED infections caused by fungi are extremely rare and are most often caused by a single pathogen, although it is estimated that it is caused by several species in 2–24.5% of cases [12, 13, 14, 15, 16, 17, 18].

## 3. Clinical Presentation

Two main clinical manifestations of infectious complications in patients with CIED may occur—pocket infection and lead-related infectious endocarditis [8]. Some authors distinguish four clinical situations: uncomplicated infection of the pulse generator, complicated infection of the pulse generator, lead infection, and infective endocarditis in a patient with CIED [2]. Symptoms of the uncomplicated pocket infection in the initial period may be scant, most often redness, swelling, and increased warmth in the area of ​​the CIED pocket. In more advanced forms, pocket abscess with purulent drainage, fistula formation, wound dehiscence, and skin erosion with externalisation of the pacemaker or leads may be observed. In an uncomplicated pocket infection, leads are not involved, the patient has no systemic signs of infection, and blood cultures remain negative. Complicated pocket infection should be diagnosed when the aforementioned symptoms are added. Systemic CIED infection is diagnosed based on systemic signs of infection, positive blood cultures, and imaging evidence of lead/valves involvement. Symptoms that should lead to the suspicion of CIED infections include fever, chills, malaise, anorexia, pulmonary embolism, and recurrent pneumonia in a patient with an implanted CIED. In imaging diagnostics, primarily transthoracic and transesophageal echocardiography and, in doubtful cases, fluorine-18-fludeoxyglucose (18F-FDG) positron emission tomography/computed tomography (PET/CT) are used [2, 8]. The European Heart Rhythm Association (EHRA) has proposed combining the modified Duke and European Society of Cardiology (ESC) 2015 criteria, based on which the final diagnosis is made [19].

## 4. Risk Factors

Risk factors for CIED infections are divided into modifiable and non-modifiable [20, 21, 22]. It should be emphasised that the first group constitutes the vast majority of risk factors, and the management of the patient in the periprocedural period should focus on their elimination. Risk factors divided into modifiable and non-modifiable are summarised in Table 1 (Ref. [20, 22, 23, 24, 25, 26, 27, 28, 29]).

**Table 1.** **Modifiable and non-modifiable risk factors for CIED infection [20, 22, 23, 24, 25, 26, 27, 28, 29]**.

Risk factors			
Modifiable	Hazard ratio	Non-modifiable	Hazard ratio
Length of procedure time	1.03–13.96	Number of previous procedures	1.03
		Number of implanted leads	5.4
Medications used:		Comorbidities:	
anticoagulants	1.08–2.8	Atrial arrhythmia	1.08–3.1
immunosuppressive therapy (i.e., glucocorticoids)	2.3–13.9	Renal dysfunction	1.5–4.8
		Dialysis	3.24–13.4
		Heart failure	3.8
		COPD	1.09–9.8
		Diabetes mellitus	2.08
		Active neoplasia	2.23
Recent fever (24 hours prior to procedure)	5.8	Complex systems (CRT-D vs. pacemaker or ICD)	1.09–1.21
Temporary transvenous pacing	1.74–2.5	Abdominal device	4.0
		Epicardial leads	8.09
Absence of preprocedural antibiotics	2.0–11.5	Younger age	1.4–1.6
Operator inexperience	2.5–2.85	Male sex	1.5–1.63
Pocket hematoma	27.2	Previous CIED (upgrade/replacement procedure)	1.56–7.84

Abbreviations: CIED, cardiac implantable electronic device; COPD, chronic obstructive pulmonary disease; CRT-D, cardiac resynchronisation therapy cardioverter-defibrillator; ICD, implantable cardioverter-defibrillator.

Several scales have been developed to objectify the risk of developing CIED infection in the future in patients undergoing CIED implantation procedures, one of which is the CIED-AI Score. The score’s name was derived from an acronym of the variables included: CIED-AI score (Charlson comorbidity Index, more than two leads/Electrodes, Device revision/replacement, oral Anticoagulation, previous Infection). Individual components were assigned specific point values that, when summed up, present the risk of CIED infection in a patient [30]. Other scales used in the prediction of CIED infections include PADIT, SHARIFF, KOLEK, MITTAL, and PACE-DRAP. Their summary is presented in Table 2 (Ref. [29, 31, 32, 33, 34, 35]).

**Table 2.** **The most frequently used scales in CIED infection risk prediction**.

Score risk	Variable	Value	Score points	Estimated infection risk
CIED-AI [31]	Charlson index >4	3	0	0.0%
	Charlson index >5	4	3	0.3%
	Three or more leads/electrodes	5	4	0.6%
	Device revision/replacement	4	5–8	0.9%
	Oral anticoagulation	5	9–10	2.5%
	Previous EI or CIED infection	8	11–17	4.1%
			>18	20.6%
PADIT [32]	<60 years	2	0–4	<1%
	60–69 years	1		
	Renal insufficiency (eGFR <30 mL/min)	1		
	Immunocompromised	3	5–6	1–3%
	ICD	2		
	CRT	4		
	Revision/upgrade	5		
	Number of previous procedures:		≥7	>3%
	1	1		
	≥2	4		
SHARIFF [33]	Diabetes	1	<3	Low
	Heart failure	1		
	Oral anticoagulation	1		
	Chronic corticosteroid use	1		
	Renal insufficiency (Cr >1.5 mg/dL)	1		
	Prior CIED infection	1	≥3	High (2.4%)
	>two leads	1		
	Epicardial leads	1		
	Temporary transvenous pacing	1		
	Generator replacement or upgrade	1		
KOLEK [34]	Diabetes	1	<2	Low
	Renal insufficiency (Cr ≥1.5 mg/dL)	1		
	Anticoagulation	1		
	Chronic cortiocosteroid use	1		
	Preimplant fever or leukocytosis	1	≥2	High (1.9–2.2%)
	Prior CIED infection	1		
	≥three transvenous leads	1		
	Pacemaker dependence	1		
	Early pocket reentry (within two weeks of implantation)	1		
MITTAL [35]	Early pocket reintervention	11	0–7	1%
	Male sex	6		
	Diabetes	3	8–13	3.4%
	Upgrade	2		
	Heart failure	1	≥15	11.1%
	Hypertension	1		
	Renal dysfunction (eGFR <60 mL/min)	1		
PACE DRAP [29]	Valvular prosthesis	2	<6	0.7%
	Hypertension (≥160/100 mmHg)	2		
	Cancer (within last five years)	2		
	Age ≥75 years	2		
	CRT/ICD	2	≥6	4.6%
	Upgrade	2		
	Clopidogrel	2		
	Ticagrelor	3		
	Renal dysfunction (eGFR <60 mL/min)	1		

Abbreviations: CIED, cardiac implantable electronic device; Cr, creatinine; CRT, cardiac resynchronisation therapy device; eGFR, estimated glomerular filtration rate; EI, infective endocarditis; ICD, implantable cardioverter-defibrillator.

A modifiable risk factor for CIED infection is the presence of temporary transvenous pacing leads before the implantation of a permanent pacemaker [21]. This solution should be reserved only for patients who do not respond to pharmacological treatment (atropine, isoprenaline, salbutamol, pressor amines) or transthoracic pacing. Moreover, temporary transvenous pacing should be used for as short a time as possible. An alternative solution is the implantation of a semi-permanent system until the active infection process is resolved. This method involves the placement of a permanent lead through the internal jugular or subclavian vein and connection to a pulse generator on the skin outside the venous access site [36, 37]. The main advantage of the aforementioned semi-permanent temporary transvenous pacing system is the active fixation of the lead, which allows for obtaining appropriate pacing parameters over a longer period of time, compared to the unstable lead for temporary transvenous pacing. The use of semi-permanent pacing, compared to temporary transvenous pacing, is also associated with a significantly lower risk of major complications [38]. In another study, it was shown that the use of this type of therapy as a bridging therapy is associated with a significantly reduced risk of late endocarditis (hazard ratio (HR) 0.25, 95% CI 0.09–0.069, *p* = 0.01) [39]. Suarez *et al*. [40] suggested that temporary transvenous pacing should be reserved only for patients who are not haemodynamically stable enough to be transferred to a fluoroscopy room. Additionally, any indwelling central venous catheters not absolutely required for further patient treatment should be removed prior to CIED implantation [32]. Interesting data were provided by the analysis of the nationwide cohort in Denmark. The authors of the analysis confirmed a significantly higher risk of complications, including infectious complications, in the case of CIED out-of-hours procedures. Therefore, these procedures should be postponed and performed during standard working hours [41].

It is worth emphasising the relationship between the duration of the procedure and infectious complications in patients with CIED. In one study, multivariate analysis showed that a procedure lasting more than 60 minutes is associated with a nearly 14-fold risk of infectious complications [29]. In other studies, a lower increase in this risk was observed, although it was still clearly elevated [26]. The longer procedure time may result from the complexity of the implanted systems as well as from the performance of procedures by inexperienced operators. It has been confirmed that when the procedure is performed by a doctor who has performed less than 100 procedures, it is associated with a nearly three-fold increase in the risk of infectious complications [22]. Undoubtedly, it is one of the modifiable risk factors of infectious complications in patients with CIED, which we can eliminate through the appropriate training of electrophysiologists. An additional opportunity to shorten the duration of the procedure is the further development of methods and devices [27]. Interestingly, not only operator experience but also hospital volume are associated with the risk of future infectious complications [42, 43].

## 5. The Use of Modern Methods of Electrotherapy in Patients at High Risk of CIED Infection

If a patient is identified as being at high risk of developing CIED (i.e., haemodialysis patients), implantation of a leadless pacemaker, epicardial device, or subcutaneous-ICD (S-ICD) should be considered if ICD implantation is necessary [44, 45, 46, 47].

### 5.1 Leadless Pacemaker

The features of the leadless pacemaker that reduce the risk of future infectious complications are the device’s smaller surface, the lack of intravascular elements, no device pocket, turbulent blood flow within the right ventricle, and subsequent device encapsulation. Perylene coating of the device provides additional protection against contamination [48]. Currently, the only leadless pacemaker approved for commercial use by the Food and Drug Administration (FDA) is the Micra Transcatheter Pacemaker System (Medtronic, Minneapolis, MN, USA), of which over 50,000 have been implanted worldwide by 2019. In clinical trials involving over 3000 patients with risk factors for subsequent infectious complications after implantation of the leadless pacemaker, not a single case of infection of the device was found [49]. To date, only four cases of leadless pacemaker infections have been published in the literature, all of which concern immunocompromised patients [50]. Importantly, in addition to a significant reduction in the risk of infectious complications, based on the meta-analysis, it was shown that the leadless pacemaker in the one-year observation provided good pacing thresholds [51]. Based on the results of the European Heart Rhythm Association survey, the main limitation of using the leadless pacemaker on a larger scale seems to be its cost and difficulty with reimbursement of the procedure, which was observed in many countries [52].

### 5.2 Subcutaneous ICD

The idea behind subcutaneous ICD (S-ICD), which is to ensure a lower percentage of infectious complications, is the lack of any elements in the vascular system. According to the recommendations of the American Heart Association (AHA), this type of device is recommended for patients with venous obstruction and those at high risk of infectious complications [53]. Secondary analysis of the PRAETORIAN trial showed that lead-related complications and systemic infections were more prevalent in the transvenous ICD group compared to the subcutaneous ICD group. In addition, complications in the first group were more severe, as they required significantly more invasive interventions [54]. Moreover, even in patients with an S-ICD implanted after removal of the transvenous ICD due to infection, the rate of future infectious complications was still low (1.3% in a three-year follow-up) [55]. The results of the S-ICD Post Approval Study gave a slightly higher percentage of infectious complications for S-ICD (3.3%). However, no bacteremia related to infection was observed. Additionally, patients who developed S-ICD infection did not have a higher mortality rate [56]. According to ESC guidelines, the subcutaneous defibrillator should be considered an alternative to a transvenous defibrillator in patients with an indication for an ICD when pacing therapy for bradycardia, cardiac resynchronisation, or anti-tachycardia pacing is not needed [57].

### 5.3 Future Perspectives

It is anticipated that Boston Scientific’s (Marlborough, MA, USA) novel “Empower” leadless pacemaker and the S-ICD will soon integrate wireless communication between devices to facilitate the coordination of leadless pacing, defibrillation therapy, and anti-tachycardia pacing, offering patients an entirely leadless equivalent to a transvenous ICD system [58]. We also expect access to the commercial use of the Aurora extravascular implantable cardioverter-defibrillator (EV-ICD) system (Medtronic, Minneapolis, MN, USA), which enables defibrillation, anti-tachycardia pacing (ATP), and backup pacing therapies without components in the patient’s venous system. Commercial access to the system is planned for 2023 [59].

## 6. Re-Implantation after CIED Removal due to Infection

For patients who have had a CIED removed due to its infection, implantation of the next device should be planned in the contralateral site or epicardially to reduce the risk of spreading infection from the prior tissue infection to the newly implanted device. Such procedures are possible with the subxiphoid approach or by using thoracoscopic tools during minimally invasive thoracotomy. Pacemakers implanted in this way are characterised by stable stimulation parameters in the mid-term [60]. A wearable cardioverter defibrillator (WCD; LifeVest WCD4000, ZOLL, Pittsburgh, PA, USA) represents a temporary alternative approach to the prevention of sudden cardiac death in patients after ICD removal. The solution enables the completion of the course of antibiotic therapy and the implantation of a permanent ICD system after completion [61]. The implantation of a leadless pacemaker in pacemaker-dependent patients undergoing transvenous lead extraction due to infectious complications seems to be an interesting solution. The effectiveness and safety of such a procedure in the mid-term were confirmed by Beccarino *et al*. [62] During a median follow-up of 163 days, no recurrence of infectious complications was found in any of the patients.

## 7. Pre-Procedural Considerations

Pre-operative preparation includes determining three basic issues: whether the patient truly has indications for a CIED implantation, whether the patient has high-risk factors for developing CIED infection, and whether the current moment is optimal for performing a CIED procedure. A key element of prevention is identifying patients at high risk for CIED infections based on the risk factors mentioned above. For this purpose, the previously presented risk scales for infectious complications can also be used. After identifying a patient as high risk, the absolute indications for CIED implantation should be reassessed, and the use of electrotherapy methods associated with a lower risk of subsequent infectious complications, such as S-ICD or leadless pacemaker, should be considered. It also seems rational in that situation to plan an early follow-up visit at the CIED implanting centre to detect possible early infectious complications—primarily pocket infection.

It is important to choose the optimal time to perform the procedure in patients during which the risk of subsequent infectious complications is lowest. To date, few studies are available on laboratory parameters that predict future infectious complications. In their multivariable analysis, Sławiński *et al*. [63] identified the elevated C-reactive protein (CRP) level at the time of cardiac implantation as the only independent predictor of the future need for an early transvenous lead extraction procedure (among others, due to CIED infections). In addition, the CIED implantation procedure should be postponed in a feverous patient. Weaker evidence is present for leukocytosis in the pre-operative period. It seems unjustified to postpone the procedure due to the presence of only isolated leukocytosis without additional accompanying symptoms of infection [64]. There is scientific evidence of a significant increase in the risk of CIED infection in the setting of pocket hematomas. The risk of pocket hematoma after CIED surgery increases significantly among patients receiving low-molecular-weight heparin (LMWH) bridging compared to continuing treatment with novel oral anticoagulants (NOACs). LMWH bridging is associated with an up to 15 times higher risk of pocket hematoma, while the risk of hematoma does not increase significantly with NOAC [65]. In addition, the continuous use of warfarin in patients at high risk of thromboembolic complications was associated with an evidently lower incidence of clinically significant pocket hematomas compared with LMWH bridging [66]. Moreover, according to the results of the randomised, double-blind, placebo-controlled trial BRIDGE, bridging anticoagulation may be of no benefit in preventing thromboembolism and may increase the incidence of bleeding [67]. For this reason, it is definitely not recommended to use bridge therapy with low molecular weight heparin [68]. Furthermore, in a large randomised trial designed by Birnie *et al*. [69], the interrupted NOAC strategy (the last dose of rivaroxaban/apixaban two days before the procedure, the last dose of dabigatran before the procedure, depending on the glomerular filtration rate) and the continued NOAC strategy (without stopping the drug, with the drug supply also in the morning on the day of the procedure) were proven to be associated with equally low rates of clinically significant pocket hematomas. To avoid pocket hematomas, in the case of elective procedures, it is recommended to postpone the procedure until dual antiplatelet therapy is discontinued, and if possible, drugs from the P2Y12 inhibitor group should be discontinued five to 10 days before the planned procedure [70]. Some authors also suggest postponing the CIED procedure until optimal glycemic control is achieved in patients diagnosed with diabetes [71].

Before the procedure, in the case of hair presence at the site of the planned incision, these should be removed using electric clippers (not razors) close to the time of surgery [9]. Additionally, the patient is recommended to wash using an antiseptic agent the day before surgery (as recommended by the Centers for Disease Control and Prevention (CDC)) [72].

## 8. Intra-Procedural Considerations

The risk of infection increases with the duration of the procedure, which often results from the implantation of more complex systems (i.e., CRT systems). Additionally, the risk of CIED infection at three months following ICD implantation is nearly 2.5-fold higher when the procedure is carried out by operators who performed only one to 10 implants per year versus those who performed 29 or more [73]. Moreover, it is important to ensure appropriate conditions in the operating theatre to minimise the risk of future infectious complications (the presence of a proper ventilation system with positive pressure in the operating room, the optimisation of air quality with filtered air, and frequent air exchanges). The number of personnel present should also be minimised to those necessary for performing the procedure, and they should use the required protective equipment [74]. According to current EHRA recommendations, to remove bacteria colonising the patient’s skin, surgical site preparation should include alcoholic chlorhexidine 2% usage, not povidone-iodine [9]. It is essential that the antiseptic be left to dry completely before incision to give sufficient time for it to be effective [70]. The routine use of solutions containing antimicrobials used for pocket irrigation does not significantly reduce CIED infection compared to saline solutions [75]. However, in their study, Kaczmarek *et al*. [76] proved that a multi-component prevention strategy involving the application of gentamicin-collagen sponge seems to significantly reduce the rate of CIED infection and to be cost-effective. This procedure has been confirmed to be feasible and safe. As mentioned, a pocket hematoma is a significant risk factor for the development of CIED infection. Hence, its prevention during the procedure is crucial. Procedures that may reduce the risk of developing pocket hematoma include meticulous cautery of bleeding sites, application of topical thrombin, irrigation of the pocket, and the use of monofilament sutures for the sub-cuticular layer. Additionally, wound pressure applied for 12 to 24 h after skin closure may be recommended [77]. A systematic review and meta-analysis by Asbeutah *et al*. [78] showed the usefulness of using antibiotic envelopes in patients with risk factors for developing CIED infection. Their use in this group of patients significantly reduced the risk of developing CIED infection in the future, while the use of envelopes in patients without CIED infection risk factors did not result in a significant reduction in the percentage of later infections [78]. Currently available envelopes release rifampin and minocycline (8 mg rifampin for medium-sized pacemaker and 11.9 mg for large pacemaker, 5.1 mg and 7.6 mg minocycline, respectively) and are fully absorbed into the body after approximately nine weeks while eluting antibiotics (the TYRX absorbable antibacterial envelope; Medtronic, Mounds View, MN, USA). Antimicrobial activity is directed against Staphylococcus aureus (both methicillin-sensitive and methicillin-resistant), Staphylococcus epidermidis, Staphylococcus lugdunensis, Acinetobacter baumannii, and Escherichia coli [26]. Minimum inhibitory concentrations within the pocket can be reached 2 h following implant and maintained for at least one week [79]. In an effort to improve cost-benefit ratios, the ration of use guided by the PADIT score is advocated [80].

## 9. Proceedings Post-Surgery

Among the post-operative factors of significant importance in increasing the risk of developing CIED infection, early re-interventions should definitely be mentioned. These should be avoided, and pocket revision should be reserved only for patients with higher dehiscence risk [27, 28]. The patient should also be advised to avoid soaking the wound until it is entirely healed after approximately a month [9].

## 10. Prophylactic Antibiotics

It has been proven that the use of antibiotics before CIED implantation significantly reduces the risk of CIED infection. Furthermore, the lack of pre-operative antibiotic prophylaxis is the strongest predictor of CIED infection [21]. The use of intravenous cefazolin has been found to significantly decrease the incidence of CIED infections when compared with a placebo (0.63% vs. 3.28%) [81]. Alternative antibiotics may be intravenous cefepime, flucloxacillin, or vancomycin (at a dose of 15 mg/kg, mainly in penicillin-allergic patients) [82]. In patients who are allergic to both cephalosporins and vancomycin, daptomycin and linezolid are options [77]. In addition to choosing the right antibiotic, it is also necessary to administer it at the right time before the procedure—the infusion of the antibiotic one hour or less before CIED implantation is suggested [83]. Repetitive dosing of antimicrobials is not recommended after skin closure, as this has not been shown to reduce the risk of subsequent CIED infection [70, 84]. In addition, the administration of topical antimicrobials after wound closure has not been shown to impact rates of CIED infection [85]. Interestingly, patients who received post-operative parenteral and post-discharge oral antibiotics had a slightly higher infection rate than those who received only pre-procedural antibiotics (1.4% vs. 0.9%, respectively) [86]. Patients with implanted complex systems, such as cardiac resynchronisation devices, may be an exception. In this group of patients, one study confirmed a lower rate of CIED infection with prolonged (five-day) post-operative antibiotic therapy [87]. Based on a survey conducted by Heart Rhythm Society (HRS) members, it was shown that antibiotic prophylaxis is significantly less frequently used in the case of subcutaneous ICD implantation (approximately 90% of respondents) and in the case of implantable loop recorder implantation (70% of respondents) [88]. In a large cohort of patients, Malagù *et al*. [89] classified them undergoing the CIED procedure as low and high risk of future CIED infection according to the Shariff score. Patients in the low risk group received only two antibiotic administrations, while those in the high risk group were treated with a prolonged nine-day protocol. An antibiotic prophylaxis based on individual stratification of infective risk resulted in a similar rate of infection between groups at high and low risk of CIED-related infection [89].

A list of suggested methods for preventing CIED infections—distinguishing between those concerning the pre-procedural period, during CIED surgery, and post-procedural period—is presented in Table 3. The document describing in detail the methods of diagnosing and treating CIED infections, which was not the purpose of this review, is the consensus of the EHRA, HRS, and several other cardiological societies. It also describes in detail the risk factors and clinical manifestations of CIED infections. This is an excellent compendium of knowledge on how to deal with this difficult disease entity, which, due to the increasing number of implanted CIEDs, will be observed increasingly more often in cardiology departments [9].

**Table 3.** **Recommendations to reduce the risk of CIED infections (details in the text)**.

Pre-procedural period	During CIED surgery	Post-procedural period
CIED infection risk assessment using one of the validated risk scales	Care should be taken to ensure appropriate conditions in the operating room	Early re-interventions should only be performed when absolutely necessary
In the case of a high risk of CIED infection, use of leadless pacing and S-ICD should be considered	The treatment, especially complex CIED systems, should be performed by an experienced operator	Avoid soaking the wound until it is entirely healed
Antibiotic prophylaxis	Surgical place preparation with alcoholic chlorhexidine 2%	
Remove hair located in the area of ​​the planned pocket using clippers	Application of gentamicin-collagen sponge should be considered, especially in patients at high risk of CIED infection	
Washing the patient’s body with an antiseptic agent the day before surgery	Optimal surgical management to reduce the risk of pocket hematoma	
Optimal control of chronic diseases, including glycemia in patients with diabetes	Consider the use of antibiotic envelopes in patients at high risk of CIED infection	
Postponing surgery in patients with fever		
Postponing surgery should be considered in patients with elevated CRP levels		
No bridging therapy with LMWH, continuation of treatment with NOAC/VKA		
If possible, discontinuation of P2Y12 inhibitors five to 10 days before the planned procedure		

Abbreviations: CIED, cardiac implantable electronic device; CRP, c-reactive protein; LMWH, low-molecular-weight heparin; NOAC, novel oral anticoagulants; S-ICD, subcutaneous implantable cardioverter-defibrillator; VKA, vitamin K antagonists.

## 11. Conclusions

Despite their relatively low incidence, CIED infections pose a significant challenge for healthcare systems. Methods of preventing this type of complication play a key role, the most important of which is periprocedural antibiotic prophylaxis. It seems that increasing access to modern methods of electrotherapy—leadless pacemakers and S-ICD—will limit the number of transvenous lead removal procedures due to CIED infections in the future.
